# Causes of death among homeless people: a population-based cross-sectional study of linked hospitalisation and mortality data in England.

**DOI:** 10.12688/wellcomeopenres.15151.1

**Published:** 2019-03-11

**Authors:** Robert W Aldridge, Dee Menezes, Dan Lewer, Michelle Cornes, Hannah Evans, Ruth M Blackburn, Richard Byng, Michael Clark, Spiros Denaxas, James Fuller, Nigel Hewett, Alan Kilmister, Serena Luchenski, Jill Manthorpe, Martin McKee, Joanne Neale, Alistair Story, Michela Tinelli, Martin Whiteford, Fatima Wurie, Andrew Hayward

**Affiliations:** 1 Public Health Data Science, Institute of Health Informatics, University College London, London, NW1 2DA, UK; 2 Collaborative Centre for Inclusion Health, Institute of Epidemiology & Health Care, University College London, London, NW1 2DA, UK; 3 National Addiction Centre, Institute of Psychiatry, Psychology & Neuroscience, King's College London, London, SE1 1UL, UK; 4 Health and Social Care Workforce Research Unit, King's College London, London, SE1 1UL, UK; 5 Community and Primary Care Research Group, University of Plymouth, Plymouth, Devon, PL6 8BX, UK; 6 Personal Social Services Research Unit, London School of Economics, London, WC2A 2AE, UK; 7 Institute of Health Informatics, University College London, London, NW1 2DA, UK; 8 NIHR Health and Social Care Workforce Research Unit, King's College London, London, SE1 1UL, UK; 9 Pathway Charity, Pathway Charity, London, NW1 2PG, UK; 10 Department of Health Services Research & Policy, London School of Hygiene & Tropical Medicine, London, WC1E 7HT, UK; 11 Tropical and Infectious Diseases, University College London Hospitals NHS Trust, London, NW1 2PG, UK; 12 Health Services Research, University of Liverpool, Liverpool, L69 3BX, UK; 13 Public Health England, London, NW9 5EQ, UK

**Keywords:** homeless health, hospital discharge, homeless healthcare, mortality, amenable mortality, data linkage

## Abstract

**Background**: Homelessness has increased by 165% since 2010 in England, with evidence from many settings that those affected experience high levels of mortality. In this paper we examine the contribution of different causes of death to overall mortality in homeless people recently admitted to hospitals in England with specialist integrated homeless health and care (SIHHC) schemes.

**Methods**: We undertook an analysis of linked hospital admission records and mortality data for people attending any one of 17 SIHHC schemes between 1st November 2013 and 30th November 2016. Our primary outcome was death, which we analysed in subgroups of 10th version international classification of disease (ICD-10) specific deaths; and deaths from amenable causes. We compared our results to a sample of people living in areas of high social deprivation (IMD5 group).

**Results**: We collected data on 3,882 individual homeless hospital admissions that were linked to 600 deaths. The median age of death was 51.6 years (interquartile range 42.7-60.2) for SIHHC and 71.5 for the IMD5 (60.67-79.0).  The top three underlying causes of death by ICD-10 chapter in the SIHHC group were external causes of death (21.7%; 130/600), cancer (19.0%; 114/600) and digestive disease (19.0%; 114/600).  The percentage of deaths due to an amenable cause after age and sex weighting was 30.2% in the homeless SIHHC group (181/600) compared to 23.0% in the IMD5 group (578/2,512).

**Conclusion**: Nearly one in three homeless deaths were due to causes amenable to timely and effective health care. The high burden of amenable deaths highlights the extreme health harms of homelessness and the need for greater emphasis on prevention of homelessness and early healthcare interventions.

## Introduction

Homelessness has increased by 165% since 2010 in England ^1^ and our recent systematic review and meta-analysis demonstrated high levels of mortality in this group across high-income countries ^2– 4^. Studies of mortality in homeless people often use relative measures such as standardised mortality ratios (SMRs), which are typically highest for death due to violence, suicide and drug overdoses. The magnitude of the SMRs reported for these causes is partly due to their rarity in the general population. The use of relative measures may understate the importance of causes of death such as respiratory disease, cardiovascular disease and cancer; which are common in the general population but may be more common among homeless people. Reliance on SMRs may therefore focus interventions on those conditions such as suicide, drug overdose, accidents and violence; at the expense of common non-communicable diseases, despite these contributing more deaths. It is therefore important to be able to measure the proportion of deaths among homeless people that are due to different causes.

A critical challenge in establishing the contribution of different causes of death to overall mortality in homeless people is the lack of routinely recorded data on homelessness in death records ^5^. In 2019 the United Kingdom’s Office for National Statistics (ONS) began to address this issue by using a variety of methods to infer homelessness, including coroners’ reports which explicitly mention homelessness, deaths registered to postcodes known to be homeless hostels, and deaths where the address was recorded as “no fixed abode” ^6^. These were used to estimate the number of deaths in homeless groups and to measure the contribution of different causes of death to overall mortality. As well as an increase in homeless deaths of 24% over the past five years, the ONS found that in 2017 the most common underlying causes of death were accidents (including drug poisoning), suicides and diseases of the liver, comprising 40%, 13% and 9% respectively. Since coroners are typically involved in investigating unexpected deaths or deaths due to external causes, the methodology may have biased ascertainment towards these external causes of death.

Hospital discharge practices for homeless patients are often inadequate, and an analysis by homeless advocacy charities in 2012 found that 70% were discharged to the street without having their health and care needs assessed ^7, 8^. In 2013, the Department of Health launched the ‘Homeless Hospital Discharge Fund’, allocating £10 million to 52 projects in the voluntary or not-for-profit sector to test different models of specialist integrated homeless health and care (SIHHC). The overall objective was to improve hospital discharge arrangements and to develop transitional intermediate (‘step-down’) care as a bridge between acute and community care. The SIHHC schemes took many forms, but the most common model was for a housing specialist to work with a patient for a limited time to improve linkage with community health services. In hospitals with high numbers of homeless patients, housing and resettlement support was often embedded as part of a specialist ‘clinically-led’ multi-disciplinary team comprising General Practitioners, nurses, therapy and social work staff in a model developed and supported by Pathway Charity ^9^. In a small number of areas, specialist residential intermediate care facilities were developed, aiming to facilitate timely discharge to a safer, more familiar environment designed to encourage supported self-management, and speed recuperation and recovery.

The existence of these schemes provided an opportunity to identify a cohort of homeless people who had been admitted to hospital by linking hospital data from patients with mortality records, and measure the relative importance of different causes of mortality. In this paper we present a sub-analysis of these linked hospital and mortality records, forming part of a broader evaluation ^10^ of the SIHHC schemes. Our analysis aims to present causes of death for people with experience of homelessness who had been admitted to hospitals in England, and to identify unmet needs by estimating the risk of death from causes that are amenable to timely and effective health care.

## Methods

The study is a population-based cross-sectional study of deaths identified through linked hospitalisation and mortality data ^10^. We present the analysis in accordance with the REporting of studies Conducted using Observational Routinely-collected Data (RECORD) statement (see Reporting guidelines) ^11^.

Eligible participants were adults over 18 years of age with one or more admissions to hospitals in England between 1st November 2013 and 30th November 2016. In a deviation from our original protocol, for this mortality analysis we only included individuals between 20 and 84 years old at death due to small numbers of deaths outside of these limits.

Two groups of individuals admitted to hospital were included in the analysis. The first group comprised of homeless individuals admitted to hospital at any one of 17 sites with a SIHHC scheme between 1st November 2013 and 30th November 2016. SIHHC schemes had various ways of identifying patients they should engage with under their remit: some relied on clinicians referring patients to them, others did ward rounds, and others undertook a combination of both strategies. This means that for the purpose of this study, our definition of “homeless” is largely defined by who the SIHHC schemes engaged with. Whilst these services focus on street homelessness and homeless people living in hostels, they may also work with those who fulfil broader definitions of homelessness such as those who are sofa surfing, or at risk of losing their existing tenancy. We conducted a comprehensive audit to identify all SIHHC hospital sites as part of a wider evaluation of the SIHHC schemes. The sample size was determined by the requirements of this wider evaluation ^10^ and required 4,076 person-years of follow up per group to detect a 10% difference in hospital readmission rates between different types of SIHHC scheme. The analysis for this wider evaluation is ongoing and we do not report results related to the sample size in this manuscript.

The second group was a random sample of individuals living in Lower Super Output Areas (neighbourhoods of approximately 1500 residents) in England in the most deprived quintile, as measured by the Index of Multiple Deprivation (IMD). These individuals were selected by NHS Digital (NHS statutory body responsible for collating and linking data across NHS datasets) and were admitted to hospitals offering SIHHC schemes during the study period, but were not seen by the SIHHC scheme (subsequently referred to as the IMD5 group). In this analysis, the IMD5 group is used to compare causes of death in homeless people with those living in socially deprived neighbourhoods. In future analyses it will be used to identify comparative cause specific mortality rates.

**Figure 1.**
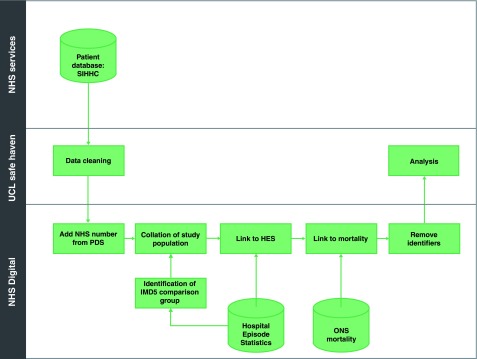
Study data flows. HES, Hospital Episode Statistics; IMD, index of multiple deprivation; NHS, National Health Service; ONS, Office for National Statistics; PDS, Personal Demographics Service; SIHHC, specialist integrated homeless health and care; UCL, University College London.

Study data flows and linkage are shown in Figure 1. For the SIHHC cohort, we collected patient identifiers collected by the services including forename, surname, date of birth and sex. Where possible, further data were also collected (aliases and NHS number - a unique ten-digit numeric identifier for patients in the healthcare system assigned at first encounter) in order to improve the linkage to Hospital Episode Statistics (HES; results not presented in this study) and Civil Registration records. These data were cleaned following some basic data cleaning principles including: removing leading/trailing spaces, recoding data to ensure consistent naming conventions (eg changing “Female” to “F”), re-formatting dates to a consistent date format. When collected, the NHS numbers were considered valid if they were 10 digits long (excluding separators) and could be validated using the modulus 11 algorithm (see NHS data dictionary) SIHHC records were then linked by NHS Digital to both HES and Civil Registration death data using NHS Digital’s “standard algorithm” (see NHS Digital page on linked data) and as described in detail in our previously published protocol ^10^. For the IMD5 group, a random sample of patients from deprived areas who had also been admitted to hospitals with a SIHHC service, and corresponding Civil Registration death records, was generated by NHS Digital. The linked data we received from NHS Digital for both SIHHC and IMD5 group was at individual person-level.

In this paper we report on the contribution of different causes of death to overall mortality in homeless people using SIHHC services, and compare these to the IMD5 group who were living in areas of high social deprivation. We categorise deaths by ICD-10 chapter ^12^; causes amenable to healthcare; and deaths that are related to suicide, drugs or alcohol (see Extended data for definitions and full ICD-10 code lists used ^13^) ^14^. We present a descriptive analysis of the causes of death in the SIHHC group compared to the IMD5 group by age, sex and ethnicity. Since the age and gender distribution of the homeless and IMD5 groups were markedly different, we weighted deaths in the homeless group by age group (at death) and sex, using the IMD5 decedents as a reference population, following the method for calculating standardised proportional mortality ratios ^15^. Where applicable, we compare our mortality data to those in the recent analysis of homeless deaths by the ONS (see Extended data for definitions and full ICD-10 code lists used ^13^). For these comparisons, we use unweighted analyses as the age and gender distribution of the homeless populations from which deaths arose was likely to be similar in the ONS analysis and our study. With the exception of ethnicity, all baseline characteristics are anticipated to be fully observed (chronic disease is presumed to be absent unless recorded). Missing values of ethnicity will be analysed grouped as ‘Not stated or not known’. We analysed data in Stata/MP version 14.2 and R version 3.5.1.

### Ethics and information governance

Collection of patient identifiers and data linkage were performed without explicit consent from participants. We undertook this research without explicit consent due to the complexities in retrospectively identifying and consenting those most likely to benefit from SIHHC services, and because we wanted to use existing secondary data for this analysis that enabled us to examine mortality in this group. We identified through user engagement with people who had experience of homelessness, that the majority felt that consent is not required for data linkage studies that aim to improve health services for homeless people, so long as adequate data security measures are used and studies are ethically and legally approved.

This research was undertaken following approval (reference 16/CAG/0021) from the Secretary of State for Health through the Confidentiality Advisory Group (CAG). Health Research Authority Research Ethics Committee approval was also sought and received (REC 16/EE/0018). In addition, local R&D approvals were set up prior to local data collection at each of the SIHHC sites. After data linkage we destroyed personal identifying data and undertook all analyses using the de-identified dataset outlined previously and in Figure 1. All study data were stored on the UCL Data Safe Haven, which has been certified to the ISO27001 information security standard and conforms to the NHS Information Governance Toolkit.

## Results

**Figure 2.**
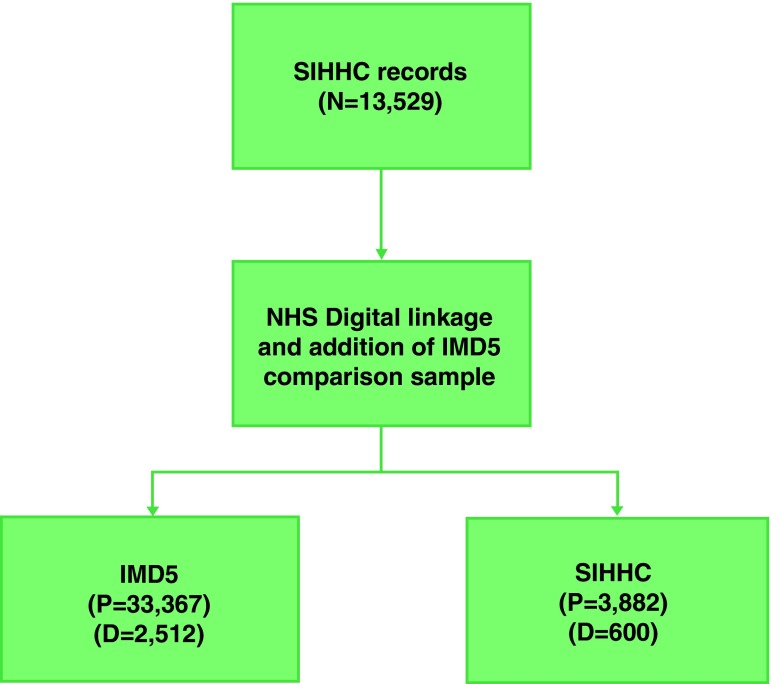
Patient Flow Diagram. N=Number of admission which multiple records for individuals, D=Number of deaths, P=number of patients using HES unique identifier.

We collected data for 3,882 individual homeless hospital admissions from 17 SIHHC sites ( Figure 2). SIHHC sites that data were collected from covered seven out of nine statistical regions in England (see Extended data ^13^) with London having the highest number of participants. Identifiable data were sent to NHS Digital for linkage and we received back Civil Registration death data for 600 in the SIHHC group and 2,512 people in the IMD5 group.

Table 1 shows the demographic characteristics of decedents in each group. Males made up 77.7% (466/600) of deaths in the SIHHC group and 56.4% (1,416/2,512) in the IMD5 group. The median age of death was 51.6 (interquartile range 42.7-60.2) for the SIHHC and 71.5 for the IMD5 group (60.67-79.0). Ethnicity was broadly similar across comparison groups with 12.9% (403/3,112) of ethnicities either not known or not stated. The top three underlying causes of death by ICD-10 chapter in the SIHHC group were external causes of death (21.7%; 130/600), cancer (19.0%; 114/600) and digestive disease (19.0%; 114/600; Table 2) in the unweighted analysis.

**Table 1.** Baseline demographic characteristics and percentage of deaths in each comparison group.

	All	IMD5	SIHHC
Total	3112 (100.0%)	2512 (100.0%)	600 (100.0%)
Sex			
Male	1882 (60.5%)	1416 (56.4%)	466 (77.7%)
Female	1230 (39.5%)	1096 (43.6%)	134 (22.3%)
Age			
20–34	269 (8.6%)	171 (6.8%)	98 (16.3%)
35–44	199 (6.4%)	84 (3.3%)	115 (19.2%)
45–54	370 (11.9%)	205 (8.2%)	165 (27.5%)
55–64	483 (15.5%)	356 (14.2%)	127 (21.2%)
65+	1791 (57.6%)	1696 (67.5%)	95 (15.8%)
Ethnicity			
British, Irish (white) and any other white	2234 (71.8%)	1792 (71.3%)	442 (73.7%)
African, Caribbean, Indian, Pakistani, Bangladeshi, Chinese and Mixed	475 (15.3%)	389 (15.5%)	86 (14.3%)
Not stated or not known	403 (12.9%)	331 (13.2%)	72 (12.0%)

IMD5 - Index of Multiple Deprivation 5 (high social deprivation), SIHHC - specialist integrated homeless health and care

*column percentages

**Table 2.** Underlying causes of death by international classification of disease 10 (ICD-10) chapter and amenable mortality.

Cause of death	Homeless (%)	Homeless weighted (%) ***	IMD5 (%)
Total	600 (100.0%)	600 (100.0%)	2512 (100.0%)
**ICD-10 chapters**			
I: Infections	18 (3.0%)	7 (1.1%)	36 (1.4%)
II: Cancers	114 (19.0%)	131 (21.8%)	926 (36.9%)
- Other	48 (8.0%)	76 (12.6%)	355 (14.1%)
- Digestive	28 (4.7%)	25 (4.1%)	242 (9.6%)
- Lung	22 (3.7%)	17 (2.9%)	247 (9.8%)
- Lymph/blood	16 (2.7%)	13 (2.2%)	82 (3.3%)
IV: Endocrine, nutritional and metabolic	17 (2.8%)	13 (2.2%)	61 (2.4%)
V: Mental & behavioural	14 (2.3%)	21 (3.5%)	82 (3.3%)
VI: Nervous system	11 (1.8%)	9 (1.6%)	94 (3.7%)
IX: CVD	96 (16.0%)	180 (30.1%)	571 (22.7%)
- Stroke	17 (2.8%)	63 (10.6%)	145 (5.8%)
- Ischaemic Heart Disease	39 (6.5%)	60 (10.1%)	251 (10.0%)
- Other	18 (3.0%)	26 (4.3%)	89 (3.5%)
- Other heart disease	22 (3.7%)	31 (5.2%)	86 (3.4%)
X: Respiratory	60 (10.0%)	100 (16.7%)	339 (13.5%)
- COPD	36 (6.0%)	52 (8.7%)	213 (8.5%)
- Other	24 (4.0%)	48 (8.0%)	126 (5.0%)
XI: Digestive	114 (19.0%)	77 (12.8%)	227 (9.0%)
- Liver	83 (13.8%)	51 (8.6%)	69 (2.7%)
- Other	31 (5.2%)	25 (4.2%)	158 (6.3%)
XX: External	130 (21.7%)	44 (7.4%)	99 (3.9%)
- Accidents	105 (17.5%)	35 (5.8%)	66 (2.6%)
- Self-harm	15 (2.5%)	6 (1.0%)	14 (0.6%)
- Other	10 (1.7%)	3 (0.6%)	19 (0.8%)
Other	26 (4.3%)	17 (2.8%)	77 (3.1%)
Deaths related to alcohol, drugs or suicide *			
Alcohol	97 (16.2%)	59 (9.8%)	67 (2.7%)
Drugs	79 (13.2%)	22 (3.6%)	23 (0.9%)
Suicide	24 (4.0%)	9 (1.5%)	22 (0.9%)
Other	400 (66.7%)	510 (85.1%)	2400 (95.5%)
Amenable to health care **			
Amenable	166 (27.7%)	181 (30.2%)	578 (23.0%)
Not amenable	434 (72.3%)	419 (69.8%)	1934 (77.0%)

IMD5 - Index of Multiple Deprivation 5 (high social deprivation), CVD – cardiovascular disease

*See extended data for ICD-10 code lists used to define underlying causes of death due to alcohol, drugs or suicide

**See extended data for ICD-10 code lists and age restrictions used to define amenable causes of death.

***Deaths in the homeless group have been weighted so that their age and sex mix matches deaths in the IMD5 group. The homeless group is younger, so weighting means that causes of death that typically occur at a younger age (e.g. accidents) receive less overall weight.

In an age and sex weighted analysis, the top three underlying causes of death in the SIHHC group were cardiovascular (30.1%; 180/600), cancer (21.8%; 131/600) and respiratory disease (16.7%; 100/600). In this weighted analysis, the underlying causes of death for each cohort by ICD-10 showed a greater proportion of deaths with an underlying cause in “External Causes of Morbidity and Mortality” chapter, in the homeless group (7.4%; 44/600) compared to the IMD5 (3.9%; 99/2,512) group. This chapter covers a range of causes including accidents, intentional self-harm, assault and events of undetermined intent (eg poisoning). Weighted alcohol and drug-related causes were higher in the SIHHC (9.8%; 59/600; and 3.6%; 22/600 respectively) than in the IMD5 group (2.7%; 67/2,512; and 0.9%; 23/2,512 respectively). The percentage of deaths with an amenable cause of mortality was higher in the homeless SIHHC group (30.2%; 181/600) compared to the IMD5 group (23.0%; 578/2,512).

**Figure 3.**
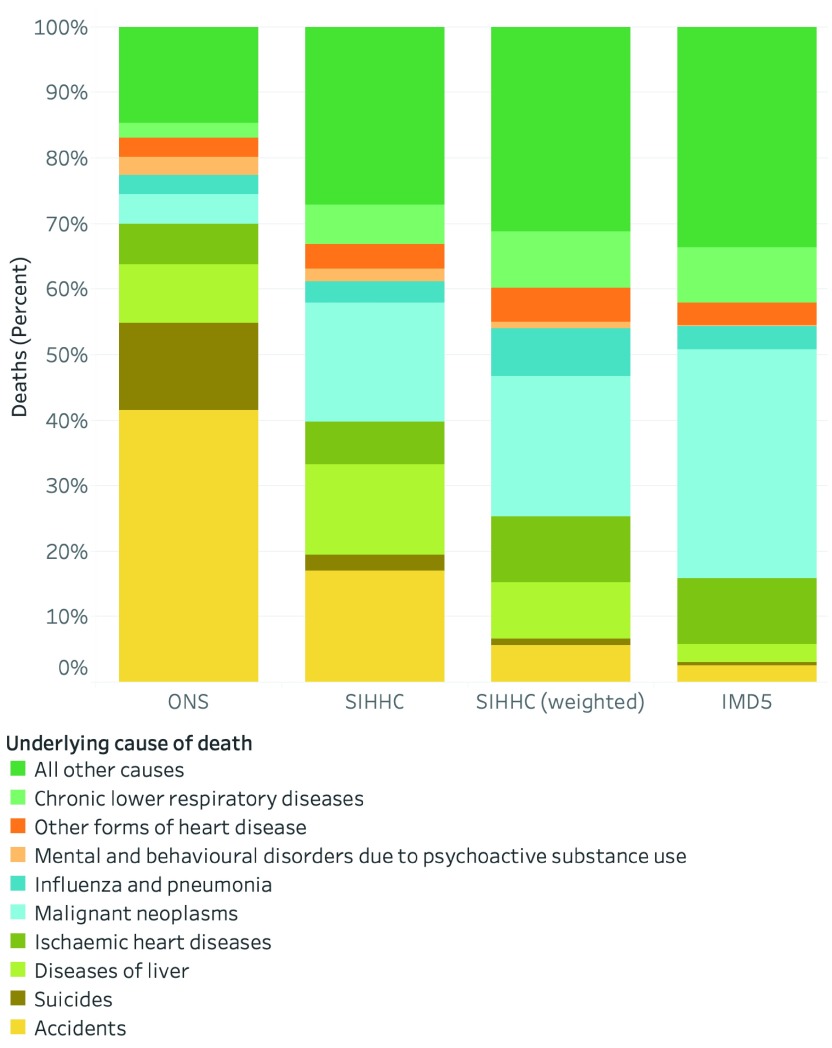
Underlying cause of death across the three comparator groups. Note: ONS causes of death estimated. See extended data file 3 for international classification of disease 10 (ICD-10) code lists used for each underlying cause of death.

In an unweighted analysis that compared the SIHHC group to the ONS homeless causes of death analysis, accidents and suicides contributed to a smaller percentage of all underlying causes of death in the SIHHC homeless group (19.5%; 117/600) compared to the ONS homeless group (54.9%; 319/581; Figure 3). Conversely, we observed a larger number of deaths due to cancer (18.2%; 109/600 vs. 4.6%; 27/581), diseases of the liver (13.8%; 83/600 vs. 9.0%; 52/581) and chronic lower respiratory disease (6.0%; 36/600 vs. 2.2%; 13/581) in the SIHCC group compared to the ONS analysis.

## Discussion

Our study investigated causes of death in a large group of homeless people admitted to hospital. We collected data on over 13,000 hospital admissions from 17 specialist sites across England, covering seven out of nine regions of the country. Our analysis confirms previous findings that external causes of death are considerably more common in homeless groups than in those from socially deprived areas. Our work also highlights the importance of causes such as coronary heart disease, respiratory disease and cancer in homeless people. Nearly one in three of homeless deaths were due to causes that are amenable to timely health care. When compared to people living in deprived areas, a greater proportion of deaths among homeless people were due to external causes (such as drugs, alcohol and suicide), and diseases of the respiratory, cardiovascular and digestive systems. Compared to the recent analysis of deaths of homeless people in England and Wales ^6^, we found that the proportion of deaths due to cancers and chronic lower respiratory disease was higher, and the proportion of alcohol, drug-related and suicide deaths was lower.

The linkage between hospital and death records enabled us to determine long-term outcomes in our groups. A further strength of our study is the certainty with which the homeless groups were identified because these individuals had been seen by services that provide support specifically for people with experience of homelessness. While “No Fixed Abode” (NFA) can be entered as a patient’s address in NHS systems, the use of this coding is inconsistent and as a result has limitations as marker of homelessness within HES records ^16^.

The deaths in this study for the SIHHC and IMD5 groups relate to people who have had a hospital admission at centres providing an SIHHC service. We were not able to identify patients accessing Primary Care services, which would be likely to provide an insight into deaths of patients with less acute health conditions but may also include unexpected deaths that occur without prior hospitalisation. The IMD5 comparison group was older and included more women; we weighted our analysis to account for these differences. For comparisons with ONS homeless mortality we used unweighted analyses, as we assumed a similar age distribution of the homeless population from whom deaths arose in both studies. We have not reported person-time based mortality rates in this study as these analyses are currently ongoing.

The higher levels of deaths due to alcohol-related causes and drug poisoning in the recent ONS data compared to our study may be as a result of the large number of death records in this ONS study identified from coroners’ reports. Our study is not subject to this bias but we recognise that deaths arising from those admitted under care of specialist homeless services may also not be representative of all deaths. We only have death data for people with experience of homelessness who were admitted to hospital, and therefore our results will exclude those people who died never having accessed healthcare - many of whom may be individuals who die due to external causes. Our results will likely over-represent people with chronic diseases admitted to hospital treatment, compared to the ONS analysis. All methods employed to measure the homeless population have limitations and it is difficult to envisage a method that would provide a fully generalisable population level estimate for this excluded population. Data linkage for further services that work with homeless people is likely to offer the best opportunities for future research.

Overall, our results confirm the need for a renewed focus on the prevention of homelessness and more robust implementation of ‘what works’ ^17^ to address deep social exclusion. While homelessness is often addressed in England by means of short-term housing related support with a focus on recovery from mental illness and substance misuse ^18^, our research points to the need for a much broader focus that is encompassing of physical health and long-term condition management, especially for more common conditions such as cardiovascular disease.

Our results show that existing specialist NHS hospital discharge schemes and community homeless health services are engaging with a population with high levels of unmet healthcare needs. One third of deaths are from conditions amenable to healthcare, highlighting a failure to intervene in a timely manner. There is a clear and urgent need to identify individuals at risk earlier, and develop models of care that enable them to engage with interventions proven to either prevent or improve outcomes for early onset chronic disease. Specialist residential intermediate care facilities that are designed to encourage safe, timely transfers, support self-management, recovery and recuperation prior to undertaking a comprehensive assessment of ongoing care and support needs are likely to form part of a comprehensive response. Equally important, are tailored approaches that take account of the lived reality of homeless people to find ways to support adherence to essential medicines ^19^, a particular challenge in this population ^20, 21^, and plausibly linked to the higher share of deaths from cardiovascular disease than in the comparator population. These new services should be informed by measures to address the lack of research on interventions to tackle chronic disorders in this population and be underpinned by robust systems to monitor the health outcomes of people who have experienced homelessness, who are at present, paying the ultimate price of extreme inequity.

## Data availability

### Underlying data

To undertake this study, collection of patient identifiers and performed data linkage were performed without explicit consent from participants. This research was therefore undertaken following approval (reference 16/CAG/0021) from the Secretary of State for Health through the Confidentiality Advisory Group (CAG). Health Research Authority Research Ethics Committee approval was also sought and received (REC 16/EE/0018). All study data were stored on the UCL Data Safe Haven, which has been certified to the ISO27001 information security standard and conforms to the NHS Information Governance Toolkit. As part of these approvals and information governance frameworks we are unable to share the underlying data for this research study. Our approval only allowed researchers involved in this specific project access for the pre-specified and approved analyses. Therefore, data collection and linkage would have to be repeated with new approvals sought by anyone wanting access to the underlying data used in this analysis.

Application for access should be directed to the CAG of the Health Research Authority. Information regarding the application process and relevant links for applications are available from the CAG website. Contact details for the relevant boards as follows:

Confidentiality Advisory Group: hra.cag@nhs.net

Health Research Authority REC: contact.hra@nhs.net

### Extended data

UCL Discovery: Causes of death among homeless people: a population-based cross-sectional study of linked hospitalisation and mortality data in England. http://dx.doi.org/10.14324/000.ds.10069223 ^13^

This project contains the following extended data:

- Aldridge_HHD Wellcome Open Mortality paper_20190226_1100_ExtndedDataFile1.pdf (Definitions and ICD-10 code lists for avoidable, amenable and preventable mortality).
- Aldridge_HHD Wellcome Open Mortality paper_20190226_1100_ExtndedDataFile2.pdf (ICD-10 codes used to define underlying causes of death due to alcohol, drugs or suicide)
- Aldridge_HHD Wellcome Open Mortality paper_20190226_1100_ExtndedDataFile3.pdf (ICD codes used to classify underlying causes of death groups in Figure 2)
- Aldridge_HHD Wellcome Open Mortality paper_20190226_1100_ExtndedDataFile5.pdf (Map of number of individuals seen by SIHHC schemes by ONS statistical region)

### Reporting guidelines

UCL Discovery: RECORD checklist for ‘Causes of death among homeless people: a population-based cross-sectional study of linked hospitalisation and mortality data in England’ http://dx.doi.org/10.14324/000.ds.10069223 ^13^

Data are available under the terms of the Creative Commons Attribution 4.0 International license (CC-BY 4.0).
