# Supplementary material for: Causes of death among homeless people: a population-based cross-sectional study of linked hospitalisation and mortality data in England
Source: Wellcome Open Res. 2019 Mar 11;4:49. [Version 1] doi: 10.12688/wellcomeopenres.15151.1 (PMC6449792; doi:10.12688/wellcomeopenres.15151.1)
Supplement: Peer review history

# Peer review: Reviewer response for version 1

**DOI:** 10.21956/wellcomeopenres.16531.r35038

**Published:** 2019-04-04

**Authors:** Glen Bramley

**Affiliations:** 1 I-SPHERE, Heriot-Watt University, Edinburgh, UK

It is good to have a chance to review this article which is one output from what looks set to be a valuable study of the Specialist Integrated Homeless Health and Care initiative across a number of English hospitals. This is timely as several studies looking at health and mortality outcomes for homeless people enabled through data linkage which are coming out at this time, supporting the upsurge of general attention to ‘inclusion health’ within the public health research and policy communities.

 The study appears to be a good example of the genre which follows established protocols for this kind of work and reporting of it. It does appear to throw up findings of interest, which may serve to alter perceptions of the nature of health problems and challenges associated with homelessness. However, I have a number of comments which may serve to temper the way the findings are interpreted and more general lessons to be learned.

1. The definition of homelessness is a thorny issue which is not directly addressed. The paper starts by stating that ‘homelessness increased by 165% since 2010 in England’, a statement that cannot be allowed to stand unchallenged. The official MoHCLG Count-Estimate data on rough sleeping rose by 165%, but this is only a tiny sub-section of the broader field of homelessness, and one whose measurement is manifestly inadequate, frequently criticised and explicitly labelled as not fit for purpose by the UK Statistics Authority. The official number of acceptances of households as statutorily homeless by local authorities has risen by 23% over this period, while a perhaps more appropriate measure of ‘Core Homelessness’ rose by 28% (Fitzpatrick et al 2019 forthcoming *Homeless Monitor England 2019[* ^1^). The paper later admits (p.4) that the SIHHC services work with a range of people including street homeless, people living in hostels, and that they may include people ‘sofa surfing’ or ‘at risk of losing their tenancy’. This failure to acknowledge the true scale of the relevant population may help to explain why ONS only found homeless deaths to have increased by 24% over five years, not 165%.
2. There are some questions lurking about the selection of hospitals/areas which participated in the SIHHC, and also about potential variations between the schemes in how they operate, particularly how patients were identified for inclusion in the scheme. The former may account for some differences between the findings of this and other studies, while the latter may account for some variance within the sample. It is possible that the latter issue may be addressed in a later phase of analysis and a separate paper.
3. There is no reference to another important comparable UK study published in June 2018, Waugh et al *Health and Homelessness in Scotland* ^2^. This study is surely an important benchmark because of its size and scope, i.e. comparison of a cohort of 345,000 people who were officially homeless over 14 years (75% of the Scottish total) with age-sex matched cohorts of similar size from both most and least deprived areas. Given the inclusive nature of Scottish Homeless legislation this captures most homeless people of all types, single and family. This study underlines that messages from both the ONS study and this SIHHC study are true: the extraordinarily high association of drug and alcohol issues with a substantial minority of homeless people, as well as the very high rates of other kinds of morbidity and health service costs affecting homeless people over and above that associated with poverty and deprivation, the so called ‘cliff edge’ following the general ‘poverty gradient’.
4. It remains concerning that there are such large differences from the ONS study (this is a further reason for looking at the Waugh et al study), with only one rather tentative explanation offered. The comparisons made are unweighted but one would suggest that wherever possible age/sex weighting should be applied.
5. It is stated in the Discussion (p.9) that ‘it is difficult to envisage a method that would provide a fully generalizable population level estimate this excluded population’, but this fails to mention both the Waugh et al study above and the new H-CLIC individual record system for homelessness in England instituted from April 2018, for which data linkage is being actively planned by MoHCLG analysts. As a result of the Homelessness Reduction Act 2017, the English statutory homelessness system will come to resemble the Scottish one to a greater degree than before, because of the stronger duty to provide prevention and relief to non-priority (i.e. single) homeless people, and the duty on other public sector bodies to refer people at risk of homelessness to local authorities. Within such a system it will also be possible to distinguish different forms and gradations of homelessness, including providing a clearer profile of cases where homelessness is combined with other key disadvantages.

I have read this submission. I believe that I have an appropriate level of expertise to confirm that it is of an acceptable scientific standard.

## References

[1] Fitzpatrick: The Homelessness Monitor: England 2019. *Forthcoming*.2019;

[2] WaughA: Health and Homelessness in Scotland' Social Research Report. *Edinburgh: Scottish Government*.2018; Reference source

# Peer review: Reviewer response for version 1

**DOI:** 10.21956/wellcomeopenres.16531.r35037

**Published:** 2019-03-14

**Authors:** Danny Dorling

**Affiliations:** 1 School of Geography and the Environment, University of Oxford, Oxford, UK

This an excellent article and the comments below are simply provided to give further information and suggestions, not as any form of criticism.

 The article begins "Homelessness has increased by 165% since 2010 in England". However, on the 31 ^st ^of January 2019, the Ministry for Housing, Communities and Local Government (MHCLG) reported that an estimated 4,677 people were now sleeping rough on any one night in England, almost three times as many as in 2010 ^1^. ^^^^These figures have long been disputed, with the true number estimated to be at least twice as high. However a wider definition of homelessness would result in an increase more similar to what the authors of this paper suggest.

 In the same timeframe, the number of families housed by local authorities in temporary accommodation rose significantly, but at a lower rate, from 50, 000 in 2010 to 78,000 in 2018. In London alone there are 225,000 “hidden homeless” people aged 16-25 arranging their own temporary accommodation with friends or family ^2^.

 This paper is welcome because it helps to dispel the current pervading narrative that places the responsibility for homelessness on the individual. The causes of homelessness do not lie with the people that it affects. Consequently, the solution to the underlying problem is not just intervention on the streets. Neither is it limited to, or likely to be much helped by, the ‘payment by results’ of ‘local social enterprises’, or the issuing of ‘social bonds’. Individual interventions may be well-meaning, but they can often be only superficially successful. 

 We know from other fields like public health that to truly use evidence to drive improvements at a population level, taking a systems approach is crucial. The fitting of gastric bands, for example, may solve obesity in individual cases, but it does not have any effect at the societal level. Obesity will not be eradicated until the whole environment is dealt with.

 When the ONS released their first estimates of the number of homeless people dying on the streets on December 20 ^th ^2018, section seven of their report was titled ‘ *Proportion of deaths of homeless people that are due to drug poisoning has increased by 51 percentage points relative to the overall number of drug deaths over five years.*’ The next day the title of that section was changed to, ‘ *Drug-related deaths of homeless people increased by 52 per cent over five years*’ ^3^. ^^^^This attention to detail and correction of a single statistic by one percentage point gives the impression that what matters most when gathering evidence is statistical exactness, and then issues such as drugs— the precise drug that lead to death is identified in individual cases. In 2018, the ONS notes that one person experiencing homelessness died from smoking cannabis, while 115 died while under the influence of opiates ^4^. The fact that somewhere a doctor noted cannabis consumption as a cause of death while homeless may well be the least useful piece of information ever released by a government funded agency.

 If this paper is to be up dated, then a more critical comparison of the ONS results with these results may be warranted. The analysts at ONS can learn from this paper, and the authors of this paper can learn from some of the ONS work on the growth of homelessness more widely that they did not cite.

 Furthermore, the ONS should not be singled out here.The same could be said of much of the literature on homelessness. A report from Housing First England ^5^ cites *"A long history of alcohol dependency, heroin and crack use and anti-social behaviour*” as the main cause of homelessness for one of its service users.

 The language used by leading sector organisations matters. Simple statements can, when repeated again and again in aggregate, frame a story, shifting focus from the causes to the symptoms of a problem. With homelessness, the emphasis is so often on how the people affected suffer from alcohol or drug misuse, have ‘high/complex needs’ or all of the above, while forgetting that the evidence suggests most people affected by homelessness never come into contact with the homelessness system, and can therefore not easily be labelled under any of these categories. They may be no different from you or me.

 Preventing homelessness in the UK requires significant reformation of housing policy. In most areas, it is currently not fit for purpose. It is not just those who are homeless who suffer as a result. Millions of others pay exorbitant rents for low-quality homes over which they have insecure rights.

 Mention of this in the paper would be useful. It might be of use to draw on evidence from the past and look at similar failures in public policy where an emphasis on the symptoms, not the causes, has prevailed. Acknowledging systemic problems is a rare occurrence in UK public policy and government often focuses on treating the symptoms of a problem for short-term gain. 

 This paper could be revised to include a paragraph or two about both these wider issues and about what happens elsewhere in Europe.

 We should recognise that almost all European countries have lower income inequality than the UK and also enshrine more tenant rights into law. Rent regulation is a vital part of that. It is the only defense against arbitrary eviction ^6^. In Germany half of all householders rent privately. Often they rent using standard leases, which permit tenants to live in a property for the duration of their lives ^7^. Rent caps are enforced to stabilise rates for new tenants, and closely monitored to ensure they don’t increase too quickly. Tenants’ groups organize to complain when landlords are not penalized for breaking the law.

 In Sweden, private-sector rent levels are set through negotiations between representatives of landlords and tenants in a very similar way to how trade unions and employers negotiate pay. In 2014, the whole of Stockholm was limited to increasing rents by only 1.12 percent as a result. In the Netherlands, monthly rental fees are fixed by government. Government officials inspect properties for quality and decide rents accordingly. Denmark has two forms of rent regulation and does not suffer homelessness on the scale of countries with a supposedly more “free market.” “Free” housing markets serve only to benefit those with most money.

 In France, a new set of rent regulations came into force in the capital in August 2015, stating that private rents “must be no more than 20 percent above or 30 percent below the median rental price for the area.” Of course, the rules prompted anger among property agencies and landlords, who claimed they would deter investment. But the evidence from less equal countries is clear: landlords charging whatever rent they choose does not result in more housing becoming available. These controls have helped reduce rent inflation as firms and European agencies move parts of their workforce to Paris during the Brexit process.

 The dominant narrative in most countries remains one that always focuses on the apparent deficits and perceived failures of people who become street homeless. But the UK is worse than most countries in this (although not as bad as the USA). Victim-blaming is an area in which much of western society excels.Thankfully, there is now growing evidence that this may be changing, and that attitudes in the UK are rapidly altering today ^8^. Changing old habits won’t be easy, but nurturing a learning sector that acts more promptly on existing knowledge and tests its assumptions about what works will improve the positive impact of our efforts. 

 This letter, one of many in recent years, was published in *The Guardian *newspaper on February 3 ^rd^2019 ^9^. It stands out because, unlike the majority of articles or obituaries written about people who have died while experiencing homelessness, Sharron Maasz was named:

*“The death of Sharron Maasz, though the subject of a coroner’s inquest, would probably otherwise have passed unnoticed. I knew Sharron well. I taught her when I was head of her middle school. Her father, a single parent, was a friend and was for a number of years a governor. Sharron was a bright, lively and sensitive girl. She was a keen cyclist and an all-round athlete. This may be her only obituary.*

*She is quoted as saying: ‘I just want to get my life sorted… I always wanted to get clean.’*

*She didn’t get sorted or clean. Instead, she died in a short-term home, a last refuge provided for those in desperate need. She had been living alone on the freezing streets of our leading university city.*

*I do not have solutions. I only know that the dreams that Sharron, a lovely child, had until her death, have perished in the wreckage of an austerity programme that has literally killed her and her like.”*

**Roger Pepworth**

*Headteacher, Marston middle school, Oxford 1983-1991*

I have read this submission. I believe that I have an appropriate level of expertise to confirm that it is of an acceptable scientific standard.

## References

[1] Ministries of Housing, Communities and Local Government: Rough Sleeping Statistics Autumn 2018, England. *[Accessed 13th March 2019]*. Reference source

[2] FranshamMDorlingD: Homelessness and public health. *BMJ*.2018; 10.1136/bmj.k214 10.1136/bmj.k214 29378686

[3] Office for National Statistics: Deaths of homeless people in England and Wales: 2013 to 2017, Section 7 'Drug-related deaths of homeless people increased by 52 per cent over five years'. Reference source

[4] Office for National Statistics: Deaths of homeless people in England and Wales: 2013 to 2017, Table 1. Reference source

[5] Housing First England: Life Stories. *[Accessed March 2019]*. Reference source

[6] HickeyS: Would a rent cap work for tenants facing £1000 a month rises?. *The Observer*.2016;

[7] DorlingD: Peak Inequality: Britain’s ticking time bomb. *Bristol: Policy Press*.2018;

[8] DorlingD: The Equality Effect. *New Internationalist (Oxford)*.2017;

[9] PepworthR: Harsh reality of life and death on the street below dreaming spires. *Letters, The Guardian*.2019;
